# Supplementary material for: Mechanisms governing the pioneering and redistribution capabilities of the non-classical pioneer PU.1
Source: Nat Commun. 2020 Jan 21;11:402. doi: 10.1038/s41467-019-13960-2 (PMC6972792; doi:10.1038/s41467-019-13960-2)
Supplement: Supplementary file 7 — Source data [file 41467_2019_13960_MOESM7_ESM.zip › Source_Data/FigureS2/FigureS2B_MotifScanOutput/KmeansCluster11/homerResults/motif12.similar.html]

motif12

## Information for motif12

C
T
A
G
A
G
T
C
C
G
A
T
A
C
T
G
G
C
T
A
A
C
G
T
C
T
A
G
G
T
C
A
  
Reverse Opposite:  

C
A
G
T
G
A
T
C
T
G
C
A
C
G
A
T
A
G
T
C
C
G
T
A
T
C
A
G
A
G
T
C
  

|  |  |
| --- | --- |
| p-value: | 1e-39 |
| log p-value: | -9.063e+01 |
| Information Content per bp: | 1.669 |
| Number of Target Sequences with motif | 1545.0 |
| Percentage of Target Sequences with motif | 47.55% |
| Number of Background Sequences with motif | 16193.6 |
| Percentage of Background Sequences with motif | 35.81% |
| Average Position of motif in Targets | 99.1 +/- 57.2bp |
| Average Position of motif in Background | 100.0 +/- 64.7bp |
| Strand Bias (log2 ratio + to - strand density) | 0.0 |
| Multiplicity (# of sites on avg that occur together) | 1.38 |
| Motif File: | file (matrix) reverse opposite |

### Similar de novo motifs found

|  |  |  |  |  |  |  |  |
| --- | --- | --- | --- | --- | --- | --- | --- |
| Rank | Match Score | Redundant Motif | P-value | log P-value | % of Targets | % of Background | Motif file |
| 1 | 0.823 | G A C T C T G A A C G T A G T C C T G A T C A G A G T C G C T A T C A G | 1e-37 | -85.246297 | 59.37% | 47.77% | motif file (matrix) |
| 2 | 0.868 | A T C G A G T C C G A T A C T G T G A C C G A T C T G A | 1e-24 | -56.353069 | 34.44% | 25.95% | motif file (matrix) |
| 3 | 0.771 | G A T C C G T A C A T G G T A C C G A T C A T G G T A C C G A T T C A G G T C A G C A T | 1e-23 | -54.292652 | 29.49% | 21.61% | motif file (matrix) |
| 4 | 0.774 | G C A T C T G A C A G T A G T C C G T A C A T G T A G C C T G A T C A G T G A C | 1e-22 | -51.647859 | 15.51% | 9.76% | motif file (matrix) |
